# A Basal Sauropodomorph (Dinosauria: Saurischia) from the Ischigualasto Formation (Triassic, Carnian) and the Early Evolution of Sauropodomorpha

**DOI:** 10.1371/journal.pone.0004397

**Published:** 2009-02-16

**Authors:** Ricardo N. Martinez, Oscar A. Alcober

**Affiliations:** Museo de Ciencias Naturales, San Juan, Argentina; University of Chicago, United States of America

## Abstract

**Background:**

The earliest dinosaurs are from the early Late Triassic (Carnian) of South America. By the Carnian the main clades Saurischia and Ornithischia were already established, and the presence of the most primitive known sauropodomorph *Saturnalia* suggests also that Saurischia had already diverged into Theropoda and Sauropodomorpha. Knowledge of Carnian sauropodomorphs has been restricted to this single species.

**Methodology/Principal Findings:**

We describe a new small sauropodomorph dinosaur from the Ischigualsto Formation (Carnian) in northwest Argentina, *Panphagia protos* gen. et sp. nov., on the basis of a partial skeleton. The genus and species are characterized by an anteroposteriorly elongated fossa on the base of the anteroventral process of the nasal; wide lateral flange on the quadrate with a large foramen; deep groove on the lateral surface of the lower jaw surrounded by prominent dorsal and ventral ridges; bifurcated posteroventral process of the dentary; long retroarticular process transversally wider than the articular area for the quadrate; oval scars on the lateral surface of the posterior border of the centra of cervical vertebrae; distinct prominences on the neural arc of the anterior cervical vertebra; distal end of the scapular blade nearly three times wider than the neck; scapular blade with an expanded posterodistal corner; and medial lamina of brevis fossa twice as wide as the iliac spine.

**Conclusions/Significance:**

We regard *Panphagia* as the most basal sauropodomorph, which shares the following apomorphies with *Saturnalia* and more derived sauropodomorphs: basally constricted crowns; lanceolate crowns; teeth of the anterior quarter of the dentary higher than the others; and short posterolateral flange of distal tibia. The presence of *Panphagia* at the base of the early Carnian Ischigualasto Formation suggests an earlier origin of Sauropodomorpha during the Middle Triassic.

## Introduction

### Basal Dinosauria

The early evolution of the Dinosauria, including the split between its two main clades Saurischia and Ornithischia, remains poorly understood because of the scarcity of fossiliferous terrestrial beds covering the late Middle Triassic (Ladinian) and early Late Triassic (Carnian). The best preserved record of Ladinian continental tetrapod assemblages comes from the Chañares Formation in Northwestern Argentina. The representative fauna includes the best known sister taxa of Dinosauria, the dinosauriforms *Marasuchus lilloensis* [1], [2] and *Pseudolagosuchus talampayensis* [3], [4]. The recently described non-dinosaurian dinosauromorphs *Dromomeron romeri* [5] and *Silesaurus opolensis* [6], found in rocks of younger (Norian) age, demonstrate that basal dinosauromorphs survived into the Late Triassic. Judging from the record of the Carnian Ischigualasto Formation assemblage (ca. 228 Ma [7]), in which the two main clades of Dinosauria has been established [8], [9], the radiation of Dinosauria, and the split of Saurischia into its two main branches, Theropoda and Sauropodomorpha, occurred in a few million years, between the sedimentation of the Ladinian Chañares and the Carnian Ischigualasto Formations.

### Carnian basal saurischians dinosaurs

Carnian basal saurischian dinosaurs are scarce and frequently fragmentary. Most are from South America, except the fragmentary *Alwalkeria maleriensis* [10] from India. The most complete and better known skeletons were found in the Carnian Ischigualasto Formation in Northwestern Argentina. This record includes *Herrerasaurus ischigualastensis* ( = *Frenguellisaurus ischigualastensis* and *Ischisaurus cattoi*), *Eoraptor lunensis* [8], [11]–[13], and three new undescribed taxa [14]–[16]. To the Carnian Upper Santa María Formation in Brazil belong the fragmentary herrerasaurid *Staurikosaurus pricei* [17] and the basal sauropodomorph *Saturnalia tupiniquim* [18]. Although *Saturnalia* presents apomorphies that confirm that it is a sauropodomorph (relatively short head, long and narrow ventral ramus of the squamosal, high tooth crowns on the anterior quarter of the tooth-bearing areas and broad distal humerus) [19], it retains morphological characters that indicate a basal position within Sauropodomorpha. Those characters are the straight dentary, fine and straight tooth serrations, tibia longer than the femur, presence of trochanteric shelf, and fourth trochanter placed proximally on the shaft of the femur, among others. Knowledge of pre-Norian sauropodomorph evolution has been restricted to this single species.

We report here a new primitive sauropodomorph dinosaur from the lower levels of the Ischigualasto Formation (Carnian) in San Juan Province, Argentina. The remains consist of one incomplete, partially disarticulated skeleton unearthed during the 2006 field season of the Museo de Ciencias Naturales of San Juan in Ischigualasto Provincial Park.

## Methods

### Preparation

The holotype was prepared using pneumatic air scribe, pin vice and water immersion. The red-brown-colored bones were embedded in a grey-green fine-grained sandstone matrix with calcareous cement. Several pieces were encased in light-grey calcareous concretions.

### Terminology

We employ traditional, or “Romerian,” anatomical and directional terms over veterinarian alternatives [20]. “Anterior” and “posterior,” for example, are used as directional terms rather than the veterinarian alternatives “rostral” or “cranial” and “caudal”. We also follow recent recommendations regarding the identification of vertebral laminae [21].

We used the phylogenetic definitions for basal taxa within Dinosauria proposed by Sereno [22]. Sauropodomorpha, for example, has a stem-based definition in opposition to Theropoda and does not require the monophyly of Saurischia or Prosauropoda (as defined, for example, by Galton and Upchurch [23]). In this way, Sauropodomorpha is defined as “The most inclusive clade containing *Saltasaurus loricatus* but not *Passer domesticus*, nor *Triceratops horridus.*”

#### Institutional abbreviations

**BRMSG** Bristol City Museum and Art Galleries, Bristol, United Kingdom.
**PVSJ** Instituto y Museo de Ciencias Naturales, San Juan 5400, Argentina.
**YPM** Peabody Museum of Natural History, Yale University, New Haven, United States of America.

## Results

### Systematic Paleontology

#### Systematic hierarchy

Dinosauria Owen, 1842

Saurischia Seeley, 1887

Sauropodomorpha Huene, 1932

***Panphagia*** **gen. nov.**

#### Etymology

*pan*, all (Greek); *phagein*, to eat (Greek); *ia*, pertaining to (Greek). In reference to the inferred omnivorous diet of the new taxon, which appears to be transitional between carnivory and herbivory.

#### Type Species

*Panphagia protos.*

### *Panphagia protos* sp. nov

#### Etymology

*protos*, first (Greek). In reference to the basal position of the new taxon within Sauropodomorpha.

#### Holotype

PVSJ 874; partial skull including the right nasal and prefrontal, left frontal, both parietals, both quadrates, right prootic, supraoccipital, anterior half of the left lower jaw, and right lower jaw lacking the anterior tip of the dentary; axial remains includes one anterior and two posterior cervical vertebrae, four posterior dorsal neural arches, one dorsal centrum, first primordial sacral vertebra, two proximal, one proximo-medial, and 15 distal caudal vertebrae; appendicular elements include the left scapula, left ilium, left pubic apron, left ischium, right tibia and astragalus, right metatarsal 3, proximal half of probable left metatarsal 4, and four pedal phalanges of uncertain position, one of which is an ungual. The bones were found disarticulated but in close association over an area of 1 m^2^. The specimen is an immature individual that has open neurocranial, neurocentral and scapulocoracoid sutures and an estimated body length of approximately 1.30 m.

#### Type Locality

**Figure 1:**
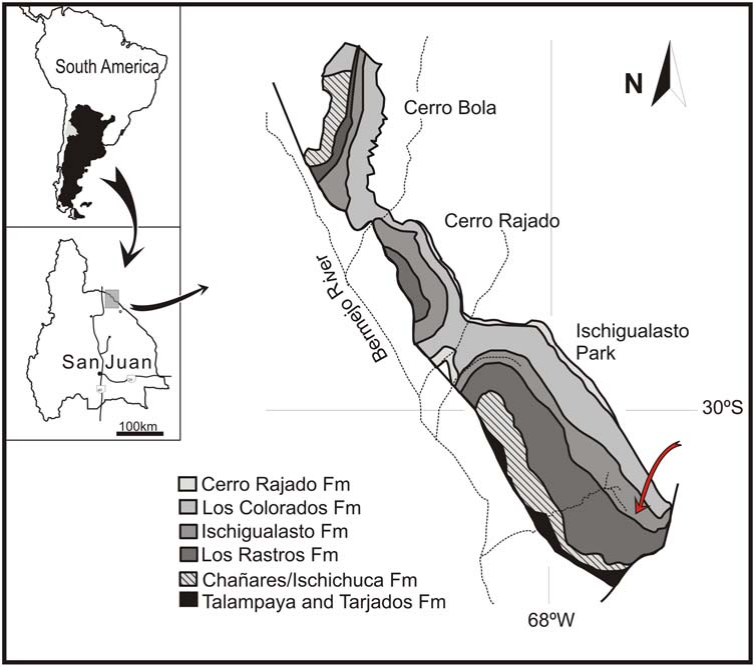
Geologic map of the Ischigualasto–Villa Unión Basin in northwestern Argentina. The red arrow points to the holotypic site of *Panphagia protos*, which is located near the base of the Carnian Ischigualasto Formation.

Valle Pintado, Hollada de Ischigualasto, Ischigualasto Provincial Park, San Juan Province, Argentina (Figure 1).

#### Horizon and Age

40 m above the base of the Ischigualasto Formation, Carnian (ca. 228.3 Mya) [7], Ischigualasto–Villa Unión Basin. The holotypic layer is approximately at the same level of the dated ash, which implies a Lower Carnian age for the specimen.

#### Diagnosis

Dinosaur characterized by an anteroposteriorly elongated fossa on the base of the anteroventral process of the nasal; wide lateral flange on the quadrate with a large foramen located far from the shaft; deep groove on the lateral surface of the lower jaw surrounded by prominent dorsal and ventral ridges, extending from the position of ninth tooth to the surangular foramen; posteroventral process of the dentary bifurcated in two slender rami that overlap the lateral surface of the angular; long retroarticular process of the articular transversally wider than the articular area for the quadrate; oval scars on the lateral surface of the posterior border of the centra of cervical vertebrae; distinct prominences located posterodorsally to the diapophyses on the neural arc of the anterior cervical vertebra; distal end of the scapula blade nearly three times wider than the neck; scapula blade with an expanded posterodistal corner limited by a wedged posterior border; and medial lamina of brevis fossa twice wider than the iliac spine.

These features distinguish *Panphagia protos* from known basal sauropodomorphs such as *Saturnalia tupiniquim* and other basal saurischians as *Eoraptor lunensis*.

### Description

**Figure 2:**
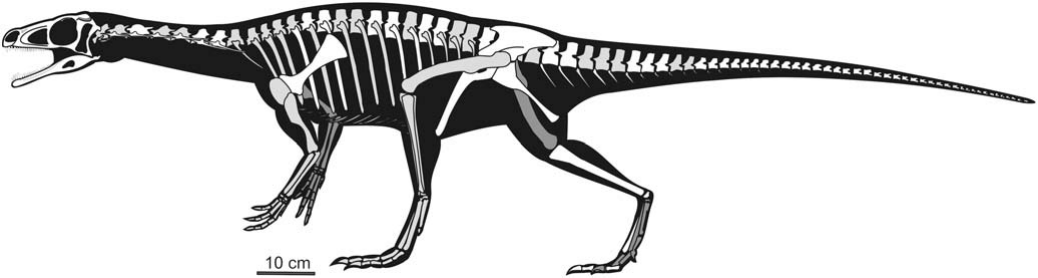
Silhouette reconstruction of the skeleton of *Panphagia protos.* Reconstruction shows preserved bones (white) and missing bones (light grey for left side; dark grey for right side). Body length is approximately 1.30 m.

Although the specimen was found in disarticulation with the exception of the 15 distal caudal vertebrae, the proximity of all pieces, the agreement in size between the different bones, and the absence of any duplicate elements all suggest these bones pertain to a single individual (Figure 2). All the limb bones and vertebrae have hollow shafts as in *Eoraptor*, *Herrerasaurus*, the basal sauropodomorph BRMSG Ca7456 [24], and neotheropods. The skeletal size and general proportions resemble those of *Eoraptor*. Nevertheless, *Panphagia* is slightly larger, axially more elongated, and has relatively shorter hindlimb bones than *Eoraptor* (Tables 1, 2).

**Table 1:** Dimensions (mm) of the axial bones of the holotypic specimen of *Panphagia protos* (PVSJ 874).

Bone	Measurements	Length
**skull**	total length of nasal	58425 (r)
	total length of frontal	43.2 (l)
	maximal width of frontal	20.3 (l)
	total length of parietal	23.55 (l)
	height of quadrate	30.4 (l); 30.85 (r)
	height of pterygoid wing	19 (l); 19.65 (r)
	transverse width of supraoccipital	17.2
	maximal length of supraoccipital	12
**lower jaw**	total length of lower jaw (estimated from both jaws)	121.05 (e)
	total length of dentary (estimated from both jaws)	73.6e
	height of dentary at mid-length	7.35 (l); 9 (r)
	anteroposterior length of external mandibular fenestra	17.6 (r)
	transverse width of quadrate facet on articular	9.15 (r)
	maximal transverse width of retroarticular process behind articular facet	9.5 (r)
	length of internal mandibular fenestra	28.35 (r)
**Vertebral column**	length of ventral contour of anterior cervical (4^th^?) centrum	25.65
	total length of anterior cervical (4^th^?)	34.85
	total height of anterior cervical (4^th^?) vertebra	22.75
	length of ventral contour of posterior cervical (7^th^?) centrum	24.6
	total height of posterior cervical (7^th^?) vertebra	25.3
	height of posterior dorsal neural arch	19
	maximal anteroposterior length of dorsal neural arch	29.625
	length of ventral contour of posterior dorsal centrum	19.825
	length of ventral contour of first primordial sacral centrum	21.85
	maximal height of first primordial sacral vertebra	45
	dorsoventral height of first primordial sacral rib iliac attachment	17.25 (l); 17.15 (r)
	maximal anteroposterior length of first primordial sacral rib	23.9

*Abbreviations*: *b*, broken; *e*, estimated; *l*, left side; *r*, right side.

**Table 2:** Dimensions (mm) of the girdle and limb bones of the holotypic specimen of *Panphagia protos* (PVSJ 874).

Bone	Measurements	Length
**scapula**	Total length	91.85(l)
	anteroposterior width of distal blade	41.5 (l,b); 45.0 (l,e)
	minimum anteroposterior width of blade neck	14.5 (l)
	maximal length from acromion to glenoid	44.9 (l)
**ilium**	Length from pubic peduncle to postacetabulkar process	46.1 (l)
	Maximum height from supraacetabular lip	15.8(l)
	Transverse width of distal end of the postacetabular process	10.1(l)
**pubis**	length of pubic apron	78.65 (l)
	mid-length width of pubic apron	24.35 (l)
	proximal width of pubic apron	26 (l)
**ischium**	total length	113.4 (l)
	length of medial lamina	39.35 (l)
	dorsoventral thickness at mid-length	7.65 (l)
	dorsoventral thickness at distal end	17.6 (l)
**tibia**	total length	157 (r)
	length of medial border of distal surface	18.15 (r)
	length of lateral border of distal surface	14.55 (r)
	length of posterior border of distal surface	18.1 (r)
**astragalus**	maximal transverse width	25.35 (r)
	transverse width of fibular facet	3.7 (r)
	anteroposterior length of medial contour in dorsal view	19.15 (r)
	anteroposterior length of lateral contour in dorsal view	15.15 (r)
	anteroposterior length of lateral border of base of ascending process	8.8 (r)
	anteroposterior length of medial border of base of ascending process	6.55 (r)
**metatarsal 3**	total length	77.3 (r)
	proximal articular surface minimal width	7 (r)
	proximal articular surface anteroposterior width	15.7 (r)
	transverse width of the distal end	12.85 (r)

*Abbreviations*: *b*, broken; *e*, estimated; *l*, left side; *r*, right side.

#### Cranium

**Figure 3:**
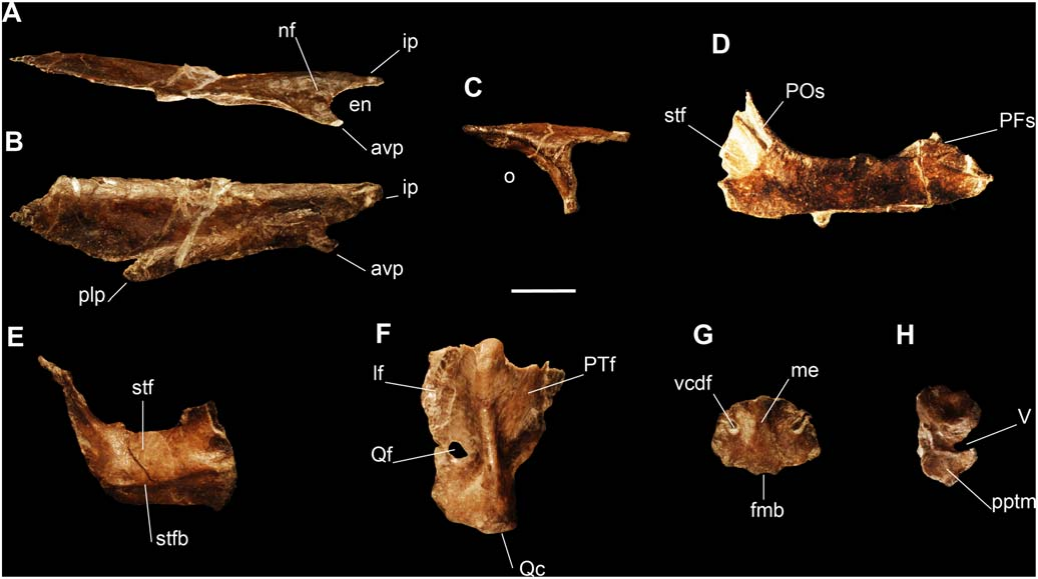
Preserved skull bones of the new basal sauropodomorph *Panphagia protos* (PVSJ 874). Right nasal in lateral (A) and dorsal (B) views. (C)-Right prefrontal in lateral view. (D)-Left frontal in dorsal view. (E)-Left parietal in dorsal view. (F)-Left quadrate in posterior view. (G)-Supraoccipital in posterior view. (H)-Right prootic in lateral view. *Abbreviations*: *avp*, anteroventral process of nasal; *en*, external nares; *fmb*, dorsal border of foramen magnum; *ip*, internarial process; *lf*, lateral flange; *me*, median eminence; *nf*, lateral fossa on nasal; *o*, orbit; *PFs*, prefrontal suture; *plp*, posterolateral process of nasal; *POs*, postorbital suture; *pptm*, M. protractor pterygoideus attachment; *PTf*, pterygoid flange; *Qc*, quadrate condyle; *Qf*, quadrate foramen; *stf*: supratemporal fossa; *stfb*, medial border of supratemporal fossa; *V*, trigeminal notch; *vcdf*, vena capitis dorsalis fossa. Scale bar equals 1 cm.

The nasal is proportionally short, measuring less than half the length of the cranium as in *Eoraptor* and basal sauropodomorphs [23] but shorter than that in *Herrerasaurus* [12] and the neotheropods “*Syntarsus*” [25] and *Coelophysis* [26]. The lateral border of the nasal is slightly concave (Figure 3B) and differs from the convex border of *Eoraptor*. The internarial process arches above the margin of the skull in lateral view (Figure 3A) as in *Eoraptor* and some basal sauropodomorphs such as *Plateosaurus* [27] and *Lufengosaurus* [28]. An anteroposteriorly elongated fossa is located on the base of the subtriangular anteroventral process (Figure 3A), which is not present in *Eoraptor* or *Herrerasaurus*. There is a well developed posterolateral process of the nasal (Figure 3B) as in *Eoraptor*, basal theropods and sauropodomorphs [19].

The prefrontal (Figure 3C) is L-shaped, with a concave orbital surface and slightly convex dorsal surface as in *Eoraptor*. The posterior process fits on a deep groove on the anterolateral surface of the frontal (Figure 3D).

The frontal is narrow between the orbits as in *Eoraptor* (Figure 3D), unlike the wide frontal of *Herrerasaurus*. The posterior part of the dorsal surface forms the anterior wall of the supratemporal fossa as in other dinosaurs [4]. The sutural surface for the anterodorsal process of the postorbital is a deep and narrow groove as in *Eoraptor* but differing from the wide sutural area of *Herrerasaurus*.

The parietal presents a slender posterolateral wing and a well marked dorsal ridge that medially delimits the supratemporal fossa (Figure 3E). The dorsal ridges of both parietals converge posteriorly but do not contact each other, unlike the condition in *Herrerasaurus*.

The shaft of the quadrate is dorsoventrally bowed as in *Eoraptor* and unlike the straight quadrate of *Herrerasaurus*. The lateral flange is transversally wider than in *Eoraptor* and *Herrerasaurus*. The quadrate foramen is large and fully enclosed in a deep fossa located on the lateral flange, above the neck, at the same level of the ventral border of the pterygoid wing (Figure 3F). This foramen is located laterally on the wing and well separated from the shaft, which is different than its position proximal on the shaft found in *Herrerasaurus*, *Eoraptor*, basal sauropodomorphs and basal neotheropods. The pterygoid flange forms more than 70% of the quadrate height as in most basal saurischians [29]. The quadrate condyle presents a well-developed sulcus of anteromedial direction, as in *Herrerasaurus* (PVSJ 53, holotype of *Frenguellisaurus*) and *Plateosaurus* [30].

The supraoccipital is much wider than it is high (Figure 3G), as in *Herrerasaurus* and basal sauropodomorphs such as *Pantydraco* [29] and *Efraasia* [31]. As in *Eoraptor*, it presents a prominent nuchal crest on the posterior surface, above the dorsal border of the foramen magnum. The foramina for the vena capitis dorsalis form deep notches on the posterior surface near the laterodorsal border (Figure 3F), similar to the non-dinosaurian dinosauriform *Silesaurus* [6].

The prootic bears a wide trigeminal notch anteromedially located and presents a well defined anteroventral surface for the protractor pterygoideus muscle (Figure 3H). Medially it preserves parts of the internal ear.

#### Lower jaw

**Figure 4:**
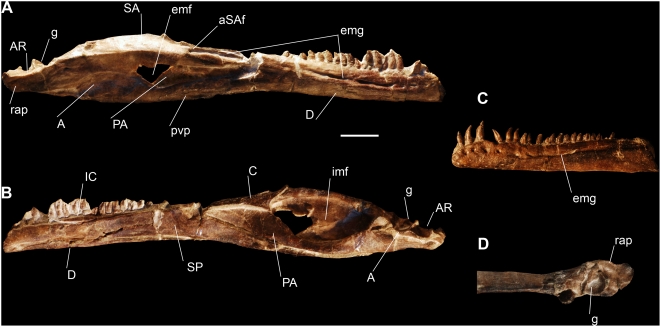
Lower jaw of the new basal sauropodomorph *Panphagia protos* (PVSJ 874). Right lower jaw in lateral (A) and medial (B) views. (C)-Fragmentary left lower jaw in lateral view. (D)-Posterior end of right lower jaw in dorsal view. *Abbreviations*: *A*, angular; *AR*, articular, *aSAf*, surangular foramen; *C*, coronoid; *D*, dentary; *emf*, external mandibular fenestra; *emg*, external mandibular groove; *g*, groove; *IC*, intercoronoid; imf, internal mandibular fenestra; *PA*, prearticular; *pvp*, posteroventral process of dentary; *rap*, retroarticular process; *SA*, surangular; *SP*, splenial. Scale bar equals 1 cm.

The lower jaw is proportionally more slender than in *Eoraptor*. The articulation of the lower jaw is located ventral to the tooth row, as in many sauropodomorphs and *Eoraptor* (Figure 4A, B). The length of the retroarticular process is greater than the depth of the mandible below the glenoid. The external mandibular fenestra is dorsoventrally pinched in its anterior portion as in *Eoraptor* and represents 16% of the mandibular length (Figure 4A). The reduction of the fenestra is also present in basal sauropodomorphs.

The dentary comprises more than 55% of the length of the lower jaw, as in *Eoraptor*, *Herrerasaurus* and many other basal saurischians. The posterior half of the ventral border of the dentary is slightly concave in lateral view, similar to that of basal sauropodomorphs such as *Pantydraco*, *Plateosaurus* and *Massospondylus* and different from the straight ventral border of *Eoraptor* and the slightly convex border of *Herrerasaurus*. The anterior part of the dentary, from the anterior tip to the level of the fourth tooth, expands dorsoventrally as in the basal neotheropods *Coelophysis* and *Syntarsus* [32] and to a lesser degree in *Herrerasaurus* and *Eoraptor*. Several large and deep neurovascular foramina open on the lateral surface of the dentary. They are located along a line parallel to the dorsal border, along the complete tooth series, but at the level of the tenth dentary tooth, they are located inside of a deep grove that posteriorly reaches the anterior surangular foramen (Figure 4A,C). Two pronounced ridges border this groove dorsally and ventrally, being the latter the most prominent. A similar groove, but limited to the dentary and without the ridges is present in *Coelophysis rodhesiensis* [26]. The ventral ridge differs from that of other sauropodomorphs as *Thecodontosaurus* neotype [24], *Plateosaurus* [30], and *Coloradisaurus* [19], in being wider at mid-length of the tooth bearing area than at the posterior end of the dentary. The posteroventral process of the dentary is bifurcated into two slender branches that overlap the lateral surface of the angular; the dorsal one reaches the ventral border of the external mandibular fenestra, and the other reaches the ventral border of the lower jaw (Figure 4A).

The splenial covers the medial aspect of the dentary and the ventral part of the intercoronoid. The mylohyoid foramen is fully enclosed by the splenial and located anteroventrally. The posterior ramus medially overlaps the ventral border of the prearticular and the anterior process of the angular. The suture between the splenial, dentary and prearticular does not show evidence of the intramandibular joint.

The surangular forms most of the lateral surface of the posterior part of the lower jaw, and the dorsal and posterior borders of the external mandibular fenestra. A small foramen opens just anterior to the posterior surangular foramen, below the surangular ridge. As in other saurischians, a large anterior surangular foramen opens anteriorly on the dorsal border of the surangular, enclosed in an anteroposteriorly oriented groove. The anterodorsal process of the surangular extends well anterior to the external mandibular fenestra, as in *Eoraptor* and unlike the short process of *Herrerasaurus* and most basal sauropodomorphs such as *Plateosaurus* [30] and *Massospondylus* [33].

The articular forms the posterodorsal border of the lower jaw. Unlike any other saurischian, the long retroarticular process is transversally wider than the articular fossa for the quadrate condyles (Figure 4D). The articular fossa is oriented along an anteromedial-posterolateral axis.

The angular extends anteriorly to its contact with the dentary and splenial at the level of the anterior border of the external mandibular fenestra. Posteriorly its distal tip is broken. Judging from the articular facet on the lateroventral border of the surangular, the slender posterior tip would have extended to the level of the retroarticular process. This long and slender posterior process of the angular is similar to that of *Eoraptor* and different from the short process of *Herrerasaurus*.

#### Dentition

**Figure 5:**
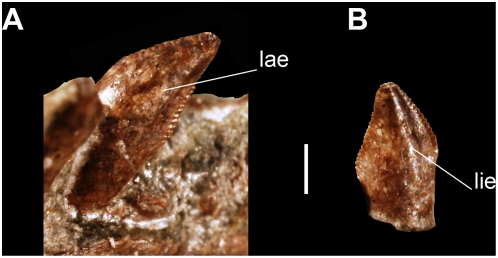
Features of the dentition of the new basal sauropodomorph *Panphagia protos* (PVSJ 874). Anterior dentary tooth in labial (A) and lingual (B) views. *Abbreviations*: *lae*, labial eminence; *lie*, lingual eminence. Scale bar equals 1 mm.

Only the dentary teeth are preserved. There are at least 23 alveoli on the left dentary (Figure 4C) and apparently 22 on the right (Figure 4A). The teeth of *Panphagia* are slightly constricted at the base (Figure 4C, 5A) as in basal sauropodomorphs and some of the teeth of *Eoraptor*. Another feature of the teeth is the presence of labial and lingual eminences that extend along the crown (Figure 5A, B). A similar eminence is present on the labial surface of the crowns of *Thecodontosaurus* neotype, and *Eoraptor*, although in the latter the lingual surfaces are unexposed. As in other basal sauropodomorphs, the teeth of *Panphagia* have coarse oblique serrations on the anterior and posterior margins (Figure 5C) that differ from the fine, perpendicular serrations present in *Saturnalia* [19] and basal saurischians. The morphology and arrangement pattern are different between the anterior quarter and the rest of the tooth series, with a fairly abrupt transition occurring after the fourth or fifth tooth (Figure 4C). This is best observed in the left rather than the right jaw, as the latter has crowns that are dislodged from their alveoli. The anterior teeth are longer than the posterior, as in *Saturnalia* [34] and most basal sauropodomorphs. They are also less basally constricted, and more posteriorly recurved. The posterior teeth are smaller, more leaf-shaped, and present more marked serrations. Although the size of each tooth is similar, the height of the crown gradually decreases backwards. They are also closely apressed in a subimbricated pattern, whereas the anterior ones are more spaced. Again, this can be best seen in the left jaw, where the teeth are in their original position.

#### Axial skeleton

**Figure 6:**
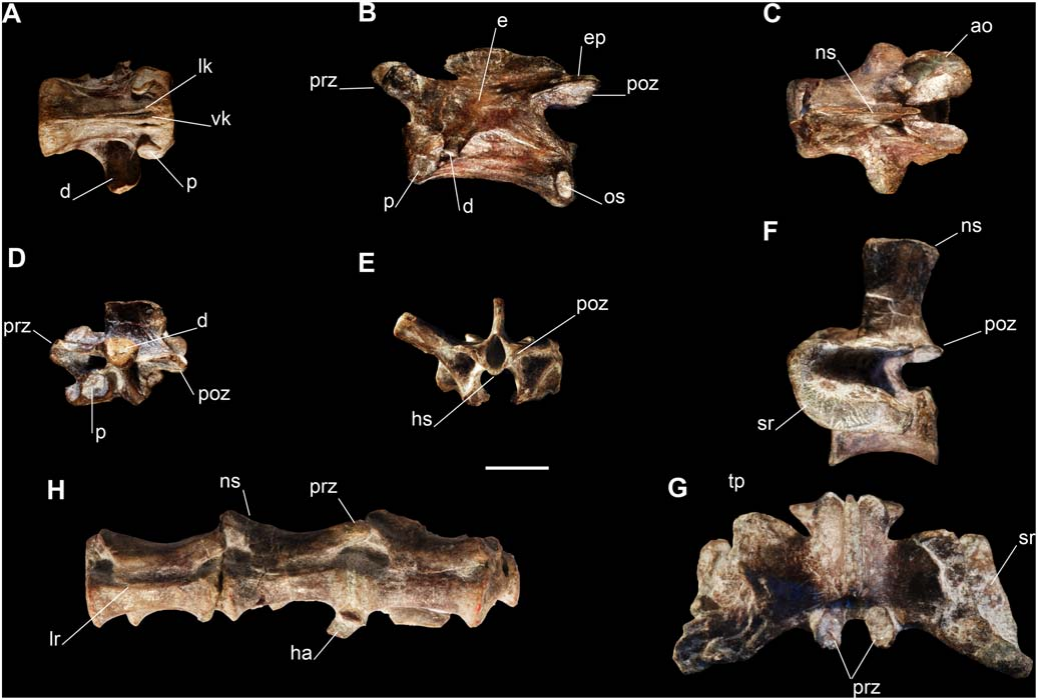
Postcranial axial skeleton the new basal sauropodomorph *Panphagia protos* (PVSJ 874). (A)-Posterior cervical vertebra (presumptive C8) in ventral view. (B)-Anterior cervical vertebra in lateral view. (C)-Posterior cervical vertebra (presumptive C7) with abnormal outgrowth in dorsal view. Dorsal neural arch in lateral (D) and posterior (E) views. First primordial sacral (S1) in lateral (F) and dorsal (G) views. (H)-Posterior caudal vertebrae in lateral view (reversed). *Abbreviations*: ao, abnormal outgrowth in prezygapophysis; *d*, diapophysis; *e*: eminence; *ha*, haemal arch; *hs*, hyposphene; *lk*, lateral ventral keels; *lr*, lateral ridge; *ns*, neural spine; *os*, oval scars; *p*, parapophysis; *prz*, prezygapophysis; *poz*, postzygapophysis; *sr*, sacral rib; *tp*, transverse process; *vk*, median ventral keel. Scale bar equals 1 cm.

Three disarticulated cervical vertebrae are preserved that likely represent C4, C7 and C8. The cervical vertebrae of *Panphagia* are slightly more elongate than those of *Eoraptor*, the neural arches being proportionately lower. The centra are parallelogram-shaped in lateral view. Their ventral and lateral sides are concave and a keel is present ventrally as in *Eoraptor*. *Panphagia* has two accessory lateroventral ridges on the anterior part of the centrum that converge posteriorly (Figure 6A). The parapophyses are located on the anterior border of the centrum. All preserved cervical vertebrae bear oval scars on the lateral surface at the posterior border of the centra (Figure 6B), a unique character for this taxon. Pleurocoels are absent as in *Eoraptor*. The neural arches are characterized by low neural spines with a convex dorsal border and a prominent, acute anterior corner. The prezygapophyses extend anteriorly farther than in *Eoraptor*. As in sauropodomorphs, the epipophyses do not extend beyond the posterior end of the postzygapophyses, unlike *Eoraptor*, *Herrerasaurus*, and neotheropods such as *Syntarsus.* The presumptive C4 has a distinct prominence located on the neural arch posterodorsal to the diapophysis (Figure 6B), which is not present in *Eoraptor* or any other basal dinosaur. The left prezygapophysis of the presumptive C7 has an abnormal bone growth that has doubled its width compared to its opposite (Figure 6C).

The cervical ribs are gracile with a long rod-shaped shafts directed posteriorly and a delicate anterior process that greatly exceeds the anterior border of the centrum as in *Eoraptor* and most saurischians. The medial surface of the cervical ribs has a deep concavity posterior to the capitulum. The tuberculum is subcylindrical and posterodorsomedially oriented. The broad capitulum is located on the medial surface of the rib and projects medially toward the parapophysis.

One centrum and four neural arches are preserved from the dorsal column of *Panphagia*. The centrum belongs to a posterior dorsal vertebra and is similar to that of *Eoraptor,* although slightly less excavated laterally. The neural arches are have well-developed laminae (prezygodiapophyseal, postzygodiapophyseal, anterior centroparapophyseal, paradiapophyseal and posterior centrodiapophyseal) that delimit deep infradiapophyseal fossae (Figure 6D). The hyposphene is dorsoventrally short, and the hypantrum is poorly developed (Figure 6E). This is similar to PVSJ 745, and *Guaibasaurus* [35] but unlike the well developed hypantrum of *Herrerasaurus* [36], *Dilophosaurus* [37] and *Massospondylus* [38], among others.

The preserved anterior (or mid) dorsal rib has a long, robust capitulum and a short, more gracile tuberculum. The capitular articular surface is larger than the tubercular surface. A small lamina spans the distance between both articular facets. The rib curves slightly distal to the union of the capitulum and tuberculum and is straight in anterior view. The posteriorly-bowed shaft has a longitudinal sulcus on its posterodorsal side that vanishes distally.

The only one sacral vertebra preserved, the first primordial sacral. The anteriorly offset, distally expanding rib is C-shaped in lateral view (Figure 6F). The posterior part of the transverse process does not reach the iliac blade (Figure 6G), as in *Eoraptor*, *Saturnalia*, *Efraasia* [39], [40], the basal sauropodomorph YPM 56733 [41], and *Plateosaurus* [42] but unlike *Herrerasaurus* and neotheropods [42].

As in the cervical series, the caudal vertebrae of *Panphagia* are proportionally longer and lower than in *Eoraptor*. The anterior transverse processes are distally expanded as in *Eoraptor*, but are posterolaterally oriented, instead of laterally as in *Eoraptor*. The prezygapophyses of the distal caudals are short (Figure 6H), unlike the condition in *Herrerasaurus* and neotheropods [36]. The hemal arches of the anterior vertebrae are long as in *Eoraptor*. The lateral surface of the posterior vertebrae presents a longitudinal ridge extending along the centrum just below the neurocentral suture.

#### Appendicular Skeleton

**Figure 7:**
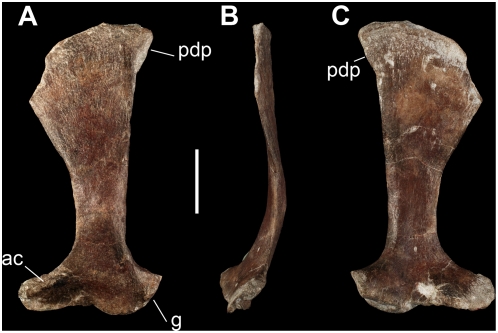
Scapula of the new basal sauropodomorph *Panphagia protos* (PVSJ 874). Left scapula in lateral (A), anterior (B), and medial (C) views. *Abbreviations*: *ac*, acromion; *g*, glenoid surface; *pdp*, posterodistal process. Scale bar equals 2 cm.

The scapula of *Panphagia* is broad and robust (Figure 7). As in *Eoraptor* [13] and *Saturnalia* [43], the proximal one-half expands gradually from the neck to the oblique dorsal borders of the acromion and glenoid. This is less derived than the abrupt right angle between the acromion and scapular blade in *Herrerasaurus*. The scapular blade is strongly expanded distally, the distal end nearly three times broader anteroposteriorly than the neck. This marked distal expansion is greater than that among other basal dinosaurs, which exhibit dital ratios of approximately two (*Saturnalia*, *Eoraptor*, *Guaibasaurus* [44]) or less than two (*Asylosaurus* [41], *Efraasia* [45]). The distal border of the blade is canted anteroventrally as in *Saturnalia*, *Guaibasaurus*, *Syntarsus* [25]. In *Eoraptor*, *Asylosaurus*, and *Herrerasaurus* [46], in contrast, the distal end is perpendicular to the long axis of the blade. The posterodorsal corner of the blade has a subtriangular extension (Figure 7A).

**Figure 8:**
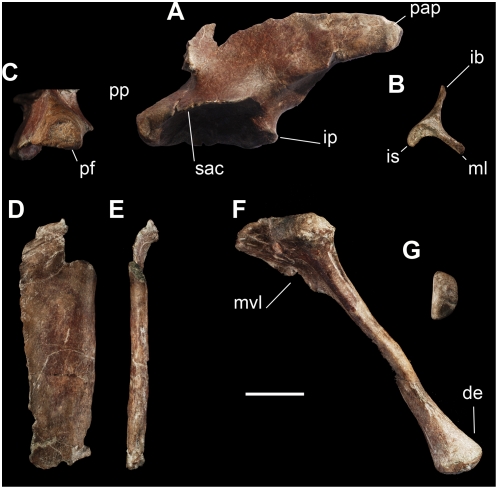
Pelvic bones of the new basal sauropodomorph *Panphagia protos* (PVSJ 874). Left ilium in lateral (A), posterior (B), and anterior (C) views. Left pubis in anterodorsal (D) and lateral (E) views. Left ischium in lateral (F) and distal (G) views. *Abbreviations*: *de*, distal expansion; *ib*, iliac blade; *is*, iliac spine; *ml*: medial lamina of brevis fossa; *mvl*, medioventral lamina; *pap*, postacetabular process; *pf*, pubic facet; *pp*, pubic process; *sac*, supraacetabular crest. Scale bar equals 2 cm.

The ilium is long and low with a well developed brevis fossa and supraacetabular crest, as in the basal saurischians *Eoraptor*, *Guaibasaurus*, and *Saturnalia* (Figure 8A). The end of postacetabular process is asymmetrical with a medial blade twice as wide as the iliac spine (Figure 8B). This condition resembles that the basal ornithischians *Scelidosaurus* and *Lesothosaurus* [19] and differs from that of *Eoraptor*, in which the brevis fossa in distal view is symmetrical. The laterodorsal surface of the postacetabular process bears a shallow depression ventral to the dorsal border; similar scars are present in some basal sauropodomorphs, such as the Mogna specimen (PVSJ 569 [47]). Although located in the same position, this scar differs from the prominent rugosity of *Saturnalia* [42] and PVSJ 845 [16]. The acetabulum is partially closed as in the basal saurischians *Guaibasaurus* and *Saturnalia*. The dorsal border of the pubic peduncle is rounded and has a semicircular cross-section (Figure 8C), differing from the triangular cross-section in *Eoraptor* with its sharper dorsal margin. The ventral border of the proximal one-half of the postacetabular process is strongly convex in lateral view, differing from the straight border in *Saturnalia*. The posterior border of the postacetabular process is slightly convex in dorsal view, similar to that in *Guaibasaurus* and differing from the concave margin that characterizes *Eoraptor* and *Saturnalia*.

Although the proximal and distal ends are lacking, the pubis seems to be elongated as in most dinosaurs, differing from the short pubis of the basal dinosauromorphs *Lagerpeton* and *Marasuchus* [48], 2 (Figure 8D–F). The pubic apron is blade-shaped with subparallel lateral and medial margins as in *Eoraptor* and most sauropodomorphs (Figure 8D). The pubic apron of *Panphagia* is anteroposteriorly straight as in *Saturnalia*, and differing from the slightly curved pubis of *Eoraptor* (Figure 8E).

The ischium is long and gracile with a medial lamina restricted to its proximal one-third as in *Eoraptor* (Figure 8F). Nevertheless, *Panphagia* has a semicircular section at mid-shaft, unlike the triangular midshaft section of *Saturnalia* and *Eoraptor*. The distal end is dorsally expanded as in *Saturnalia* [42], differing from the slightly expanded distal end of *Eoraptor* and *Herrerasaurus*. In distal view the distal end presents a semicircular outline, similar to that of neotheropods [19], but different from the triangular shape present in *Herrerasaurus*, *Saturnalia* and more derived sauropodomorphs (Figure 8G).

**Figure 9:**
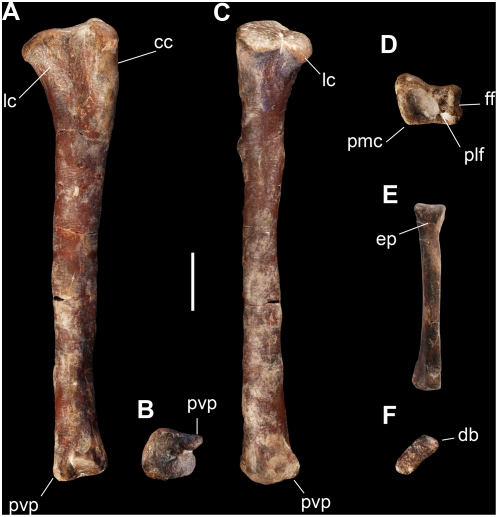
Hind limb and pedal elements of the new basal sauropodomorph *Panphagia protos* (PVSJ 874). Right tibia in lateral (A), distal (B), and posterior (C) views.(D)-Right astragalus in proximal view. Right metatarsal 3 in anterior (E) and proximal (F) views. *Abbreviations*: *cc*, cnemial crest; *db*, dorsal border; *ep*, extensor pit; *ff*, fibular facet; *lc*, lateral condyle of proximal tibia; *plf*, posterolateral fossa on proximal surface; *pmc*, posteromedial corner; *pvp*, posteroventral process. Scale bar equals 2 cm.

The tibia is similar to that of other basal saurischians such as *Eoraptor*, *Herrerasaurus*, and *Saturnalia*. The proximal end is subtriangular, the cnemial crest projects slightly anteriorly, and the distal end has a short posterolateral process (Figure 9A). The lateral condyle is located close to the posterior border in lateral view, similar to that in *Eoraptor* but differing from the more centered condyle of *Saturnalia*, PVSJ 845 [16], and more advanced sauropodomorphs. The descriptive terms used to differentiate these shapes in lateral view, however, are poorly differentiated. The distal end is subrectangular with the transverse width slightly greater than its anteroposterior length (Figure 9B). This condition is more strongly expressed in more advanced sauropodomorphs, such as *Massospondylus* [38], *Plateosaurus* [49], *Riojasaurus* [50] and others. As in *Saturnalia*, the anteroposterior length of the distal end of the tibia is greater medially than laterally, and the posterior border is slightly concave in distal view. This latter condition is similar to that in *Plateosaurus* but different from the straight or slightly convex border in *Eoraptor* and *Herrerasaurus*. The medial tip of the posterolateral process is distally short (Figure 9C), as in *Eoraptor*, and unlike the distally projected process of *Saturnalia*, *Herrerasaurus*, basal sauropodomorphs and basal neotheropods as *Syntarsus* [32].

The astragalus is subrectangular in proximal view, although the anteromedial corner is particularly prominent (Figure 9D). This condition resembles that in *Saturnalia* but differs from the more rounded anteromedial corner in *Eoraptor*. Although the ascending process is broken, the base is subrectangular and broader laterally than medially with the long axis anteroposteriorly oriented as in *Eoraptor* and *Saturnalia*. The fibular facet is transversally narrow as in *Eoraptor*, *Saturnalia*, and other sauropodomorphs. The elliptical fossa located behind the ascending process is well delimited by a ridge as in *Eoraptor*, *Saturnalia* and other basal sauropodomorphs.

Metatarsal 3 is 50% of the length of the tibia, similar to that in *Eoraptor* and *Herrerasaurus* [36]. The proximal end has a parallelogram shape in proximal view with a uniform transverse width (Figure 9F). This shape is similar to that in *Herrerasaurus* and *Eoraptor* but differs from the subtriangular shape of *Saturnalia* and other basal sauropodomorphs. The shaft is gently bowed medially (Figure 9E). The distal condyles are asymmetrical, the lateral condyle protruding laterally with a deeper extensor pit and collateral ligament fossa.

The three non-ungual pedal phalanges of uncertain position are preserved. These may well be penultimate, or at least distal, because of the presence of well developed posterodorsal processes. All have deep extensor and collateral pits, and asymmetrical condyles, resembling those of *Eoraptor*. The preserved pedal ungual phalanx is gently curved, and presumably belong to digit III, judging for its size and the symmetrical lateral grooves.

## Discussion

### Comparative Considerations

*Panphagia protos* exhibits features that place it among dinosaurs, such as a wide temporal fossa on the frontal, reduction of the external mandibular fenestra; epipophyses on postaxial cervical vertebrae, first sacral rib anteriorly expanded, and well developed brevis fossa on the ilium. It exhibits notable saurischian characters as well, such as the long mid-cervical ribs that are subparallel to the neck, a hyposphene-hypantrum articulation in the dorsal vertebrae, enlarged sacral transverse processes, a broad supraacetabular crest, and a medial lamina of the ischium restricted to the proximal one-third of the bone. In addition *Panphagia* presents a distally expanded ischium, a eusaurischian character.

Although *Panphagia* is structurally close to the common ancestor of Theropoda and Sauropodomorpha, several unequivocal synapomorphies indicate that *Panphagia* is a basal sauropodomorph. These include an enlarged external naris, concave ventral border of the dentary (lateral view), tooth size differentiation along the tooth row, sublanceolate crowns that have a slight basal constriction and oblique, coarse serrations, imbricate arrangement of posterior dentary teeth, separation between the iliac blade and the posterior part of the transverse process of the first sacral vertebra, and a fibular facet on the astragalus that is transversally narrow in dorsal view. In addition, *Panphagia* presents some other ambiguous features that strengthen a sauropodomorph affinity.

These include reduction of the external mandibular fenestra, rudimentary lateral ridge below the tooth row on the lateral surface of the dentary, lengthening of cervical vertebrae, pubic apron blade-shaped with subparallel lateral and medial margins, and distal end of the tibia subrectangular with a transverse width slightly greater than the anteroposterior length. Furthermore, the tibia of *Panphagia* is of similar length to that of *Eoraptor,* although all other bones are longer. Considering that the femoral length is correlated with the body mass [51], we can infer that the femur of *Panphagia* is relatively longer than in *Eoraptor* and that the femur/tibia ratio of *Panphagia* would have been greater than in *Eoraptor*. This also suggests a closer affinity affinity with Sauropodomorpha than with other basal dinosaur clades. Nevertheless it is possible than the entire hindlimb of *Panphagia* was relatively short compared with *Eoraptor*.

The new specimen shares some features with *Saturnalia,* such as the anteroventral inclination of the distal border of the scapular blade, dorsally expanded distal end of the ischium, lateral border of the astragalus anteroposteriorly wider than the medial border, acute posteromedial angle of the astragalus in proximal view, and ascending process of the astragalus subrectangular with long axis anteroposterior and broader laterally than medially. However, the lack of knowledge of cranial and axial elements of *Saturnalia* precludes further comparisons.

The relatively long skull of *Panphagia* represents the primitive condition when compared with the reduced skull length in other sauropodomorphs. Although the femur is unknown, we infer a skull/femur length ratio of approximately 0.7 based on *Eoraptor*. This ratio is greater than that inferred for *Saturnalia* [19], although the incompleteness of the skull and dentary of the latter casts doubt on this value.

Several features are shared between *Panphagia* and *Eoraptor*, such as the extremely hollow bones, similar structure and proportions, internarial process arched above the margin of the skull in lateral view, sublanceolate teeth with lateral prominences on the crowns, dorsoventral compression of the anterior part of the external mandibular fenestra; transverse process of the first primordial sacral vertebra not reaching the ilium, pubic apron blade-shaped with a subparallel lateral and medial margins; distal end of the tibia subrectangular with transverse width slightly greater than the anteroposterior length, medial tip of the posterolateral process of the tibia not distally projected, fibular facet of the astragalus transversally narrow; and ascending process of the astragalus subrectangular with the longer axis anteroposteriorly oriented and laterally wider than medially, among others.

The shared landmarks with *Saturnalia* are not surprising, because *Saturnalia* is currently recognized as a sauropodomorph, but the resemblance with *Eoraptor* is noticeable, especially if it is considered as a theropod [13]. Although the problem of the phylogenetic position of *Eoraptor* exceeds the purpose of this study, as was noted before, *Eoraptor* exhibit some features that resemble sauropodomorphs. Those characters are: lanceolate teeth; enlarged external nares; and mandibular joint well below the tooth row [19]. In addition several other characters can be cited: ventral ramus of the squamosal more than five times longer than anteroposteriorly wide; transverse process of the first primordial sacral vertebra not reaching the ilium; reduced olecranon on the ulna; short and pointed preacetabular process; fibular facet of the astragalus transversally narrow; ascending process of the astragalus anteroposteriorly wide; and anteroposterior length of the medial border of the astragalus in proximal view notably wider than that of the lateral border. Moreover, some of the “theropod” characters of *Eoraptor*, such as the extremely hollow limb bones are also present in basal sauropodomorphs (e.g., Mogna specimen PVSJ 610; BRMSG Ca7456 [24]), suggesting this is a plesiomorphic condition for Saurischia.

### Phylogenetic Position

In order to determine the phylogenetic position of *Panphagia protos* within basal Dinosauria, we decided to add *Panphagia* to the data matrix published by Langer and Benton [19], because it is a recent study that includes both *Saturnalia* and *Eoraptor*. To that analysis we added a line of character states for *Panphagia* (Table 3). We maintained the original character states for all the taxa except *Eoraptor*, for which we corrected several character state scores (Table 3).

**Table 3:** Character state scores for *Panphagia* protos (PVSJ 874) and *Eoraptor* lunensis (PVSJ 512).

*Panphagia*	????? ??11? ????? ??000 11010 201?? 0?1?? ?110? ??110 00???
	????? ????? ????? ???12 1100? 10011 0???1 11010 ?111? ???
*Eoraptor*	01110 11111 111?0 1?0?0 ?1000 100?? ?1110 ?1100 101?? 0?110
	02??1 0011? 11011 010?2 11110 00010 01111 10011 01?10 ??0

Data lines inserted into the data matrix of Langer and Benton [19].

**Figure 10:**
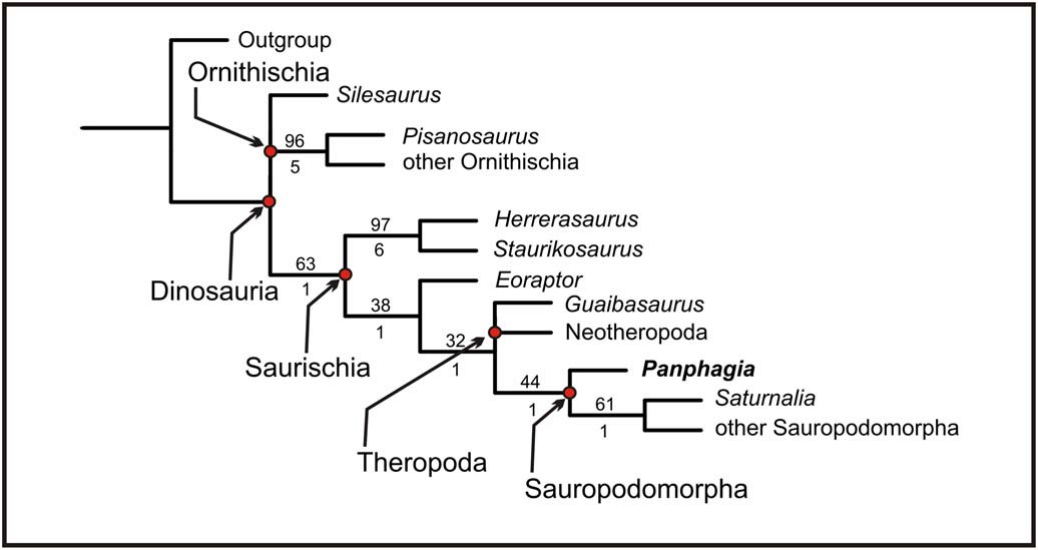
Consensus tree. Consensus of three most-parsimonious trees resulting from the present parsimony analysis (tree length 187 steps; consistency index 0.561, retention index 0.568). The jackknife frequency (p = 0.36′ 1000 replications) and Bremer support values for each node are depicted above and below the internal branch leading to that node, respectively.

We swapped “Other Ornithischia” and “Other Sauropodomorpha” for of “Ornithischians” and “Sauropodomorpha,” respectively, following the phylogenetic definitions for these taxa proposed by Sereno (2005). The new analysis resulted in three most-parsimonious trees of 187 steps (consistency index 0.561, retention index 0.568)(Figure 10). An implicit enumeration search [52] and jackknifing (probability of character removal 0.36, 1000 resampled matrices) were performed.

All of the most-parsimonius trees nested *Panphagia* within Sauropodomorpha as the most basal sauropodomorph and sister group to *Saturnalia* and other sauropodomorphs. These trees differ from those of Langer and Benton [19] in the unresolved position of *Silesaurus* and *Guaibasaurus* (Figure 10).

Four synapomorphies unite *Panphagia* with *Saturnalia* and other sauropodomorphs: dentary tooth crowns constricted at the base (character 24); lanceolate crowns in most dentary teeth (char. 26); tooth crowns of the anterior quarter of the dentary series higher than the others (character 28); and short posterolateral flange of distal tibia (character 90). The characters that place *Panphagia* as less derived than *Saturnalia* are: posteriorly curved crowns (character 25); roughly semicircular distal outline of ischium (character 81); and lateral condyle of tibia posteriorly located (character 85). The aforementioned discussion suggests that *Panphagia* represents a new distinctive sauropodomorph, representing the most primitive known taxon of Sauropodomorpha.

### Early Origin of Sauropodomorpha

The early evolution of saurischians dinosaurs from a small cursorial ancestor [13], [19] seems to be confirmed by the recent discoveries of new basal dinosaurs. With the exception of the Herrerasauridae, all of these basal forms are small-bodied species less than 3 m in length, such as *Eoraptor*, *Saturnalia* and *Guaibasaurus*. *Panphagia* and two new but unpublished basal saurischians from the Carnian Ischigualasto Formation [15], [16] are also small-bodied species. The general similarity among all of these basal dinosaurs suggest that few structural changes stand between *Eoraptor*, *Panphagia* and the new basal theropod PVSJ 560 [15]. Size increase does not appear to have been a major factor during Carnian times in this region of Pangaea.

The basal sauropodomorph *Saturnalia* was discovered in the rhynchosaur biozone of the Carnian Santa María Formation in Brazil, strata widely regarded as contemporary to the Ischigualasto Formation in Argentina based on faunal similarities [53], [54]. The absolute age of 228 My (earliest Carnian) for a level 20 meters above the base of the Ischigualasto Formation [7] suggests that deposition of the formation may have begun during the Ladinian. The presence of *Panphagia* near the base of the Ischigualasto Formation suggests that the origin of Sauropodomorpha occurred during the Ladinian or earlier during the Middle Triassic. *Panphagia* lived with at least other five different basal dinosaurs (*Eoraptor*, *Herrerasaurus*, PVSJ 605 [14], PVSJ 560 [15], and PVSJ 845 [16]) in the lower section of the Ischigualasto Formation, suggesting that saurischian dinosaurs were already well diversified at the dawn of the Carnian.
